# LAAEV-centric atrial failure phenotype and a novel integrative score for predicting early recurrence after AF ablation

**DOI:** 10.3389/fcvm.2026.1769676

**Published:** 2026-04-15

**Authors:** Rui Wang, Jing Lv, Ruixue Guo, Qian Liu, Lianxia Wang, Jidong Zhang

**Affiliations:** 1 Department of Cardiology, The Second Hospital of Hebei Medical University, Shijiazhuang, China; 2 Department of Cardiology, Xingtai People’s Hospital Affiliated to Hebei Medical University, Xingtai, China; 3 Department of Cardiology VIII, The Second Hospital of Hebei Medical University, Shijiazhuang, China; 4 Department of Cardiology I, The Second Hospital of Hebei Medical University, Shijiazhuang, China

**Keywords:** atrial fibrillation, atrial myopathy, catheter ablation, early recurrence, left atrial appendage emptying velocity, predictive score

## Abstract

**Background:**

Early recurrence of atrial tachyarrhythmia (ERAF) after radiofrequency catheter ablation (RFCA) for atrial fibrillation (AF) remains a major clinical challenge. While left atrial diameter (LAD) is a known predictor, the role of atrial mechanical function, particularly left atrial appendage emptying velocity (LAAEV), and its integration with other markers needs clarification.

**Methods:**

This single-center retrospective cohort study included 157 drug-refractory non-valvular AF patients undergoing first-time RFCA. Among them, 53 (33.8%) developed ERAF within the 90-day blanking period. Baseline clinical, echocardiographic (including LAD and LAAEV), and biochemical (NT-proBNP) data were collected. The primary endpoint was ERAF (atrial tachycardia/flutter/fibrillation ≥30 s) within the 90-day blanking period. Predictive models were developed using logistic regression. An integrative AF Recurrence Index (AF-RI) was derived and validated internally.

**Results:**

Among 157 patients, 53 (33.8%) experienced ERAF. The ERAF group exhibited a distinct “atrial failure” phenotype: larger LAD (43.8 ± 5.6 vs. 39.5 ± 4.3 mm, *p* < 0.001), lower LAAEV (35.8 ± 9.9 vs. 47.6 ± 11.9 cm/s, *p* < 0.001), and higher NT-proBNP (median 356.9 vs. 207.2 pg/mL, *p* = 0.023). LAAEV was a stronger independent predictor (AUC: 0.812, 95% CI: 0.742–0.882) than LAD (AUC: 0.745, 95% CI: 0.665–0.825). The AF-RI, integrating LAD, LAAEV, and NT-proBNP, demonstrated outstanding discrimination (AUC: 0.917, 95% CI: 0.874–0.960), with significantly higher sensitivity (88.7%) and specificity (84.6%) than single-parameter models (all *p* < 0.05). Correlation network analysis positioned LAAEV as a central hub linking structural and neurohormonal remodeling. Patients stratified by LAAEV tertiles showed dramatically graded ERAF risks (14.8%, 28.3%, 58.5%, *p*-trend < 0.001).

**Conclusion:**

LAAEV is a potent, independent predictor of ERAF, central to an “atrial failure” phenotype. The integrative AF-RI score provides a simple, bedside-friendly tool for individualized risk stratification, enabling clinicians to identify high-risk patients (e.g., those with LAAEV < 35 cm/s) who may benefit from intensified monitoring or tailored therapeutic strategies post-ablation.

## 1 Introduction

Atrial fibrillation (AF) is the most common sustained cardiac arrhythmia worldwide, posing a significant burden on healthcare systems due to its associations with stroke, heart failure, and increased mortality (1). AF is increasingly recognized as a highly heterogeneous syndrome rather than a single uniform disease entity, with marked differences in clinical presentation, underlying substrate, comorbidity burden, and prognosis across patient populations. Recent artificial intelligence-based phenotyping studies have further demonstrated that AF can be subdivided into clinically meaningful phenotypes in both general and critical care settings, supporting the need for more refined and individualized risk stratification (2).

Radiofrequency catheter ablation (RFCA) has emerged as a pivotal therapeutic strategy for patients with symptomatic, drug-refractory AF, offering superior rhythm control compared to antiarrhythmic drug therapy alone (3). Despite technological and procedural advancements, a substantial proportion of patients experience early recurrence of atrial tachyarrhythmia (ERAF) within the 90-day post-ablation “blanking period,” which serves as a strong harbinger of long-term procedural failure (4). Accordingly, improving pre-procedural identification of patients at high risk of recurrence has become increasingly important for tailoring surveillance intensity, rhythm monitoring, and post-ablation management strategies (5).

The pathophysiology underlying ERAF is multifactorial, involving a complex interplay of structural, electrical, and functional remodeling of the left atrium (LA) (6). Beyond conventional structural and electrical remodeling, growing evidence suggests that AF is closely linked to atrial cardiomyopathy, a broader pathological process involving metabolic dysregulation, mitochondrial dysfunction, oxidative stress, and progressive atrial fibrosis. Experimental data further indicate that impaired mitochondrial energy metabolism and enhanced oxidative stress may actively accelerate diabetes-related atrial remodeling and increase AF susceptibility (7).

Left atrial diameter (LAD), a conventional echocardiographic marker of structural remodeling, has been consistently linked to AF recurrence (8). However, LAD provides limited insight into atrial mechanical function, which may deteriorate independently of chamber size (9). In contrast, left atrial appendage emptying velocity (LAAEV), assessed via transesophageal echocardiography (TEE), directly reflects atrial contractile reserve and has recently gained recognition as a sensitive indicator of atrial myopathy (10). Meanwhile, emerging mechanistic work has implicated ferroptosis-related redox injury as another potential contributor to AF initiation and progression, highlighting that the biological substrate of AF extends well beyond simple chamber enlargement alone (11).

Concurrently, elevated levels of N-terminal pro-B-type natriuretic peptide (NT-proBNP) signify atrial wall stress and neurohormonal activation, further contributing to the arrhythmogenic substrate (12).

Recent studies have individually highlighted the prognostic value of LAD (13), LAAEV (14), and NT-proBNP (15) for post-ablation outcomes. Nevertheless, an integrative model that synergistically combines these readily accessible parameters to enhance risk stratification for ERAF remains elusive. This unmet need is consistent with the broader challenge in AF research that, despite major advances in mechanistic understanding, translation into truly mechanism-based therapeutic or prognostic tools remains limited (16).

Most existing predictive scores are either overly complex for bedside application or fail to incorporate functional and biochemical dimensions of atrial disease (17, 18). Currently, several predictive scores have been developed for AF recurrence after ablation. The APPLE score (Age, Persistent AF, imPaired eGFR, Left atrial diameter, EF) and the DR-FLASH score (Diabetes, Renal dysfunction, persistent Form of AF, Left atrial diameter, Age, female Sex, Hypertension) primarily focus on clinical factors and structural remodeling, but do not incorporate direct measures of atrial mechanical function (17). The MB-LATER score (Male, Bundle branch block, Left atrial diameter, Type of AF, Early recurrence) includes early recurrence as a predictor, which limits its pre-procedural applicability (18). While these scores have contributed to risk stratification, they share common limitations: reliance on indirect markers of atrial dysfunction, exclusion of functional parameters such as LAAEV, and absence of neurohormonal markers like NT-proBNP that reflect atrial wall stress. These gaps underscore the need for an integrative, pre-procedural tool that captures the multifaceted nature of atrial myopathy—combining structural, functional, and biochemical dimensions into a clinically actionable score.

Therefore, we conducted this retrospective cohort study with the following objectives: (1) to evaluate and compare the independent predictive value of LAD and LAAEV for ERAF; (2) to develop and internally validate a novel, user-friendly integrative score—the Atrial Fibrillation Recurrence Index (AF-RI)—that incorporates LAD, LAAEV, and NT-proBNP; (3) to elucidate the interrelationships between these variables through correlation network analysis; and (4) to assess the consistency of the LAAEV effect across key clinical subgroups.

## 2 Methods

### 2.1 Study design and ethics approval

This was a single-center, retrospective cohort study conducted at *the Second Hospital of Hebei Medical University*. A retrospective cohort design was chosen for this study as it allows for the efficient evaluation of multiple baseline predictors—including echocardiographic and biochemical markers—in relation to a well-defined clinical outcome (ERAF) within a real-world setting. This design is particularly suited for the exploratory phase of developing and internally validating an integrative risk score, leveraging existing clinical data to generate hypotheses and inform the design of future prospective studies.

Consecutive patients who underwent first-time RFCA for atrial fibrillation between August 2023 and October 2024 were screened for eligibility. The study protocol was reviewed and approved by the Institutional Ethics Review Board of the Second Hospital of Hebei Medical University (2022-R134). Given the retrospective, observational nature of the study, which involved the analysis of de-identified data collected during routine clinical care, the requirement for written informed consent was formally waived by the Ethics Committee. All patient data were anonymized prior to analysis to protect confidentiality. The study was conducted in accordance with the ethical standards of the Declaration of Helsinki and followed the Strengthening the Reporting of Observational Studies in Epidemiology (STROBE) guidelines for cohort studies.

We acknowledge the inherent limitations of the retrospective design, including the potential for selection bias due to the exclusion of patients with incomplete data, and the inability to establish causal relationships. However, stringent inclusion and exclusion criteria were applied to create a relatively homogeneous cohort, and multivariable analysis was performed to adjust for measured confounders. These findings should be considered hypothesis-generating and warrant validation in large-scale, prospective, multicenter cohorts.

The study retrospectively identified 183 consecutive patients who underwent first-time RFCA for atrial fibrillation. After excluding 26 patients based on pre-defined criteria (valvular AF, prior ablation, left atrial thrombus, severe concomitant heart disease, active systemic illness, incomplete data), the final analysis cohort comprised 157 adult patients with drug-refractory, non-valvular AF (NVAF).

### 2.2 Patient selection and criteria

Patients were included if they were: (1) ≥18 years old, (2) diagnosed with symptomatic NVAF refractory to at least one Class I or III antiarrhythmic drug (failure defined as recurrent symptomatic AF or drug intolerance), and (3) undergoing first-time RFCA.

Exclusion criteria were: (1) valvular AF (moderate/severe mitral stenosis or mechanical valve), (2) prior AF ablation, (3) left atrial thrombus on pre-procedural TEE, (4) severe structural heart disease (e.g., hypertrophic cardiomyopathy, LVEF < 35%), (5) active malignancy or systemic inflammatory disease, (6) end-stage renal disease (eGFR < 15 mL/min/1.73 m² or dialysis), and (7) incomplete baseline data or loss to follow-up within the 90-day blanking period.

These exclusion criteria were deliberately chosen to ensure a homogeneous study population and to isolate the specific contribution of left atrial myopathy to ERAF risk. Patients with valvular AF were excluded because the hemodynamic and pathophysiological mechanisms driving atrial remodeling in this population differ fundamentally from those in non-valvular AF, where left atrial appendage velocity may have distinct prognostic implications. Patients with prior AF ablation were excluded to avoid the confounding effects of ablation-induced atrial scarring and altered atrial mechanics on both LAAEV measurement and recurrence risk. Exclusion of patients with left atrial thrombus was mandatory for procedural safety and also serves to remove a confounder associated with severely depressed LAA function. Patients with severe structural heart disease or active systemic illness were excluded to minimize competing risks and non-arrhythmic contributors to clinical outcomes. Finally, requiring complete baseline data ensures the integrity of the multivariable models and the derivation of the AF-RI score. Consequently, the findings of this study are primarily generalizable to patients with non-valvular AF undergoing first-time ablation who have preserved or moderately reduced ventricular function and no significant concomitant valvular or systemic disease.

### 2.3 Data collection and retrospective Variable definition

All data were systematically extracted from the hospital's integrated electronic medical record (EMR), echocardiography reporting system, and the electrophysiology laboratory database by two trained investigators using a standardized form. Variables collected included: (1) demographics: age, sex, body mass index (BMI); (2) AF characteristics: type (paroxysmal or persistent, defined based on the longest documented episode within 6 months pre-ablation, with persistent AF defined as sustained >7 days or requiring cardioversion), and AF duration (calculated from the date of first documented ECG/Holter diagnosis to the ablation date); (3) comorbidities: hypertension, diabetes mellitus, coronary artery disease, prior cerebral infarction; (4) lifestyle factors: smoking and alcohol history; (5) biochemical parameters measured within 7 days pre-procedure after overnight fast: serum creatinine, uric acid, lactate dehydrogenase, low-density lipoprotein cholesterol (LDL-C), and N-terminal pro-B-type natriuretic peptide (NT-proBNP, measured via electrochemiluminescence immunoassay, Roche Diagnostics); (6) pre- and post-procedural medications: anticoagulants (warfarin, dabigatran, rivaroxaban/edoxaban) and antiarrhythmic drugs (β-blockers, propafenone/amiodarone/dronedarone). The CHA₂DS₂-VASc score was calculated for each patient.

The dataset was initially reviewed for completeness. Among the 183 screened patients, 26 were excluded due to incomplete baseline data or loss to follow-up, as specified in the exclusion criteria. In the final analysis cohort of 157 patients, there were no missing data for the primary predictors of interest (LAD, LAAEV, NT-proBNP) or the primary outcome (ERAF), as these variables were mandatory fields in the institutional electronic medical record and echocardiography reporting system. For covariates with occasional missing values (e.g., LDL-C, uric acid), which were <5% of cases, multiple imputation by chained equations (MICE) with 5 imputations was performed under the missing at random (MAR) assumption. Sensitivity analyses comparing complete-case analysis and imputed datasets yielded consistent results, supporting the robustness of the findings.

### 2.4 Echocardiographic assessment

Comprehensive transthoracic and transesophageal echocardiography was performed for all patients within 30 days prior to ablation using a standardized protocol (Philips EPIQ CVx). Key measurements included: (1) LAD measured in parasternal long-axis view at end-systole; (2) LAAEV measured via pulse-wave Doppler during TEE with the sample volume placed 1 cm inside the appendage orifice—the highest positive velocity from five consecutive cycles was averaged for patients in AF, while measurement was timed to the *P*-wave peak for those in sinus rhythm; (3) left ventricular end-diastolic diameter (LVEDD); and (4) left ventricular ejection fraction (LVEF) calculated using the biplane Simpson's method.

All measurements were performed offline by two experienced echocardiographers (R.W. and R.G.) who were blinded to clinical outcomes and patient baseline characteristics. To assess inter-observer reliability, both observers independently measured LAAEV, LAD, and LVEF in a random subset of 30 patients. To assess intra-observer reliability, the primary observer (R.W.) repeated the measurements in the same 30 patients after a 4-week interval, blinded to prior results. Intraclass correlation coefficients (ICC) with 95% confidence intervals were calculated using a two-way random-effects model for absolute agreement. The inter-observer reliability was excellent for LAAEV (ICC: 0.92, 95% CI: 0.87–0.95), LAD (ICC: 0.90, 95% CI: 0.84–0.94), and LVEF (ICC: 0.88, 95% CI: 0.81–0.93). Intra-observer reliability was also excellent for LAAEV (ICC: 0.94, 95% CI: 0.89–0.97), LAD (ICC: 0.92, 95% CI: 0.86–0.95), and LVEF (ICC: 0.90, 95% CI: 0.84–0.94). These findings confirm the reproducibility and robustness of the echocardiographic measurements used in this study.

### 2.5 Ablation procedure and post-procedural management

All RFCA procedures were performed by senior electrophysiologists. Pulmonary vein isolation (PVI) using wide-area circumferential ablation guided by a 3D electroanatomic mapping system (CARTO 3) was the standard approach. Additional substrate modification (e.g., linear ablation, complex fractionated atrial electrogram ablation) was performed for persistent AF at the operator's discretion. Procedural endpoint was electrical isolation of all pulmonary veins confirmed by entrance/exit block. Post-procedure, patients received continuous telemetry monitoring for ≥24 h. Anticoagulation was resumed on the procedure day and continued for at least 2 months. Antiarrhythmic drugs were typically maintained through the 90-day blanking period.

### 2.6 Follow-up protocol and endpoint definition

All patients were enrolled in a structured follow-up program with visits at 1, 3, 6, and 12 months post-ablation. Each visit included a 12-lead ECG. Protocol-mandated 24 h Holter monitoring was performed at 3, 6, and 12 months. Additional monitoring (7-day Holter or event recorder) was used for symptomatic episodes. The 90-day post-ablation period was defined as the blanking period. The primary endpoint was early recurrence of atrial tachyarrhythmia (ERAF), rigorously defined as any documented episode of atrial tachycardia, flutter, or fibrillation lasting ≥30 s occurring between day 1 and day 90 post-procedure (excluding the first 24 h). All rhythm tracings were independently reviewed by two cardiologists blinded to baseline data.

### 2.7 Statistical analysis

Statistical analyses were performed using R (version 4.3.1) and SPSS (version 27.0). Continuous variables are presented as mean ± SD or median [IQR] based on normality (Shapiro–Wilk test) and compared using Student's t-test or Mann–Whitney *U*-test. Categorical variables are expressed as *n* (%) and compared using Chi-square or Fisher's exact test. Univariable logistic regression identified predictors of ERAF; variables with *p* < 0.10 entered multivariable logistic regression with backward stepwise selection.

The AF-RI was developed as a simplified bedside score based on the multivariable logistic regression model. Although NT-proBNP lost statistical significance after adjustment, it was retained due to its biological relevance and contribution to model discrimination. The final formula was derived by transforming regression coefficients into simple arithmetic operations:

$$AF-RI=(LAD/10)+(40/LAAEV)+log_{10}(NT-proBNP/100)$$

where LAD is in mm, LAAEV in cm/s, and NT-proBNP in pg/mL. The constants were chosen to scale each component to comparable magnitudes while preserving the direction of effect: LAD/10 converts mm to a single-digit number; 40/LAAEV uses 40 cm/s as a clinically relevant threshold, yielding higher values for lower velocities; log₁₀(NT-proBNP/100) normalizes the skewed distribution. This simplified score retained excellent discriminative performance (AUC 0.917) compared to the full regression model (AUC: 0.924, *p* = 0.48), confirming that simplification did not compromise accuracy.

Model discrimination was assessed using area under the receiver operating characteristic curve (AUC), compared by DeLong's test. Calibration was evaluated via Hosmer-Lemeshow test. Decision curve analysis (DCA) assessed clinical utility.

To assess the internal validity of the predictive models and quantify optimism, we performed bootstrap internal validation with 1,000 resamples. For each bootstrap sample (drawn with replacement from the original cohort of 157 patients), the entire model development process—including variable selection and coefficient estimation—was repeated. The apparent performance (AUC in the bootstrap sample) and test performance (AUC when applying the bootstrap-derived model to the original dataset) were computed. The optimism was calculated as the mean difference between apparent and test performance across all bootstrap samples. The optimism-corrected AUC was then derived by subtracting the optimism from the original model's apparent AUC.

Model calibration, the agreement between predicted probabilities and observed outcomes, was assessed using two complementary approaches. First, the Hosmer-Lemeshow goodness-of-fit test was performed, with a *p*-value > 0.05 indicating no significant departure from perfect calibration. Second, a calibration plot was generated, plotting observed vs. predicted probabilities across deciles of risk, accompanied by a loess-smoothed calibration curve. The calibration intercept and slope were calculated, with an ideal model having intercept = 0 and slope = 1.

To further guard against overfitting, we employed several strategies: (1) Parsimonious variable selection: Only variables with *p* < 0.10 in univariable analysis were entered into the multivariable model, and backward stepwise selection with Akaike Information Criterion (AIC) was used to retain only the most informative predictors; (2) Events per variable (EPV): With 53 outcome events and a final model containing 2–3 predictors, the EPV exceeded 15, well above the minimum recommended threshold of 10 to ensure stable coefficient estimates [HARRELL2001]; (3) Simplified scoring: The AF-RI was derived using clinically intuitive transformations (division by 10, reciprocal, log10) rather than regression coefficients, which inherently reduces the risk of coefficient overfitting to the specific dataset; (4) Sensitivity analysis: We compared the performance of the full regression-based model and the simplified AF-RI, confirming minimal performance degradation (AUC: 0.924 vs. 0.917), indicating that the simplification did not compromise predictive accuracy.

Kaplan–Meier analysis with log-rank test compared recurrence-free survival by LAAEV tertiles. The association between LAAEV decrease (per 10 cm/s) and ERAF odds was evaluated across prespecified subgroups using logistic regression with interaction terms. Prespecified subgroup analyses were performed to assess the consistency of the association between LAAEV (per 10 cm/s decrease) and ERAF odds across key baseline subgroups, including age (<60 vs. ≥60 years), sex, AF type (paroxysmal vs. persistent), and the presence of hypertension. A two-tailed *p*-value < 0.05 was considered statistically significant.

## 3 Results

### 3.1 Study population and baseline characteristics

This single-center retrospective cohort study enrolled 157 consecutive adult patients with drug-refractory NVAF who underwent first-time RFCA. Within the 90-day blanking period, 53 patients (33.8%) experienced ERAF of atrial tachyarrhythmia (any episode ≥ 30 s), constituting the ERAF group, while 104 patients (66.2%) remained recurrence-free (non-ERAF group).

A comprehensive comparison revealed the cohorts were well-matched in demographics, most comorbidities, lifestyle, and routine biochemistry (Table 1). However, patients with ERAF had a higher prevalence of persistent AF (43.4% vs. 30.8%, *p* = 0.027), a longer AF duration (median 29.5 vs. 23.8 months, *p* = 0.037), and exhibited a phenotype of advanced atrial disease: significantly large LAD (43.8 ± 5.6 vs. 39.5 ± 4.3 mm, *p* < 0.001), markedly reduced LAAEV (35.8 ± 9.9 vs. 47.6 ± 11.9 cm/s, *p* < 0.001), and higher NT-proBNP levels (median 356.9 vs. 207.2 pg/mL, *p* = 0.023).

**Table 1:** Baseline clinical, echocardiographic, and biochemical characteristics.

Characteristic	Total (*N* = 157)	ERAF + (*n* = 53)	ERAF- (*n* = 104)	*p*-value
Demographics				
Age, years	59.9 ± 10.5	59.8 ± 11.3	59.9 ± 10.1	0.955
Male, *n* (%)	117 (74.5)	39 (73.6)	78 (75.0)	0.898
BMI, kg/m²	26.0 ± 3.5	26.3 ± 4.1	25.8 ± 3.1	0.356
AF profile				
Persistent AF, *n* (%)	55 (35.0)	23 (43.4)	32 (30.8)	0.027
AF Duration, months	25.0 [7.0, 58.0]	29.5 [11.8, 83.5]	23.8 [5.3, 47.5]	0.037
Comorbidities, *n* (%)				
Hypertension	80 (51.0)	28 (52.8)	52 (50.0)	0.67
Diabetes Mellitus	27 (17.2)	9 (17.0)	18 (17.3)	0.956
Coronary Artery Disease	26 (16.6)	11 (20.8)	15 (14.4)	0.246
Prior Cerebral Infarction	12 (7.6)	4 (7.5)	8 (7.7)	0.993
Lifestyle, *n* (%)				
Smoking History	66 (42.0)	24 (45.3)	42 (40.4)	0.486
Alcohol History	75 (47.8)	29 (54.7)	46 (44.2)	0.155
Biochemical Parameters				
Creatinine, μmol/L	78.2 ± 14.3	76.6 ± 12.3	79.3 ± 15.5	0.185
Uric Acid, μmol/L	370.0 [298.0, 420.0]	388.9 [301.5, 413.7]	356.2 [296.2, 427.5]	0.505
Lactate Dehydrogenase, U/L	158.0 [139.5, 186.0]	154.5 [140.7, 179.6]	160.5 [138.9, 190.3]	0.468
NT-proBNP, pg/mL	250.0 [89.5, 685.0]	356.9 [133.7, 914.2]	207.2 [69.6, 622.5]	0.023
LDL-C, mmol/L	2.54 ± 0.75	2.55 ± 0.78	2.53 ± 0.73	0.745
Echocardiographic Parameters				
LAD, mm	41.0 ± 5.3	43.8 ± 5.6	39.5 ± 4.3	<0.001
LVEDD, mm	56.9 ± 3.5	57.9 ± 3.8	56.3 ± 3.4	0.112
LVEF, %	48.5 ± 3.6	47.1 ± 3.9	49.3 ± 3.1	0.445
LAAEV, cm/s	43.2 ± 12.3	35.8 ± 9.9	47.6 ± 11.9	<0.001
Post-procedural medication, *n* (%)				
Warfarin	2 (1.3)	1 (1.9)	1 (1.0)	0.894
Dabigatran	137 (87.3)	43 (81.1)	94 (90.4)	0.07
Rivaroxaban/Edoxaban	18 (11.5)	9 (17.0)	9 (8.7)	0.065
*β*-blockers	70 (44.6)	27 (50.9)	43 (41.3)	0.168
Propafenone/Amiodarone/Dronedarone	98 (62.4)	36 (67.9)	62 (59.6)	0.18
Risk score				
CHA₂DS₂-VASc Score	1 [0, 2]	1 [0, 2]	1 [0, 2]	0.94

Data are mean ± SD, median [IQR], or *n* (%). BMI, body mass index; LAD, left atrial diameter; LVEDD, left ventricular end-diastolic diameter; LVEF, left ventricular ejection fraction; LAAEV, left atrial appendage emptying velocity; LDL-C, low-density lipoprotein cholesterol.

The ERAF group exhibited a 4.3 mm larger left atrial diameter (43.8 vs. 39.5 mm), which exceeds the threshold considered indicative of clinically relevant atrial remodeling (>40 mm) and represents a 10.9% relative increase. More strikingly, LAAEV was reduced by nearly 12 cm/s (35.8 vs. 47.6 cm/s), a 25% relative reduction, moving from the normal range (>45 cm/s) into the severely impaired range (<40 cm/s) associated with a 3-fold increased risk of thromboembolism and arrhythmia recurrence in prior studies. The median NT-proBNP level was 72% higher (356.9 vs. 207.2 pg/mL) in the ERAF group, exceeding the 250 pg/mL threshold previously linked to adverse outcomes post-ablation. The 9.6-month longer median AF duration (29.5 vs. 23.8 months) and 12.6% higher prevalence of persistent AF (43.4% vs. 30.8%) further characterize the ERAF group as having a more advanced and hemodynamically consequential atrial myopathy. These clinically meaningful differences collectively define an “atrial failure” phenotype that is readily identifiable at the bedside using routine pre-procedural assessments.

Clinically relevant thresholds for key parameters based on prior literature: LAD > 40 mm indicates significant atrial enlargement; LAAEV < 40 cm/s indicates severely impaired atrial mechanical function and is associated with increased thromboembolic risk and AF recurrence; NT-proBNP > 250 pg/mL has been associated with adverse outcomes post-ablation.

### 3.2 Temporal distribution and predictive performance of individual parameters

**Figure 1:**
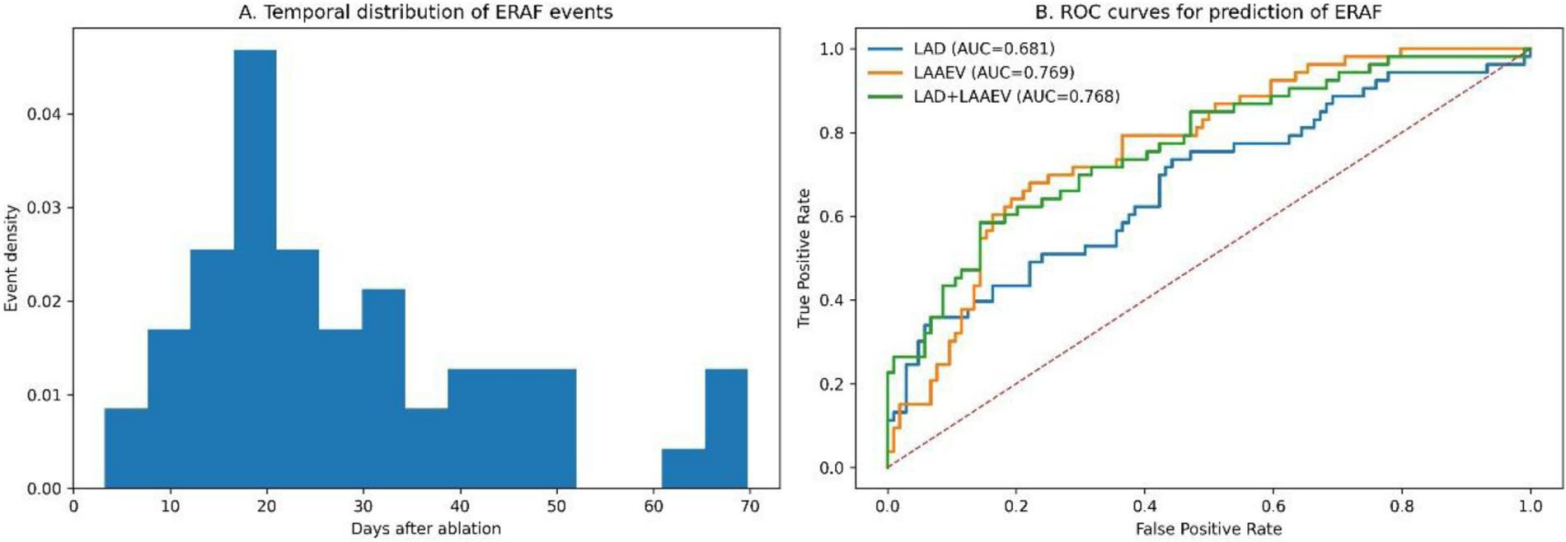
Temporal dynamics and predictive performance. **(A)** Density curve of ERAF events over the 90-day blanking period. **(B)** Receiver operating characteristic (ROC) curves for LAD, LAAEV, and their combined logistic regression model.

The temporal distribution of ERAF events during the 90-day blanking period revealed a distinct pattern (Figure 1A). A kernel density estimation plot demonstrated a prominent peak in the early post-ablation phase, with the highest event density occurring within the first 30 days, followed by a gradual decline and sporadic occurrences in the later phase of the blanking period. This pattern aligns with the clinical understanding of early recurrence dynamics following catheter ablation.

The predictive performance of key echocardiographic parameters was evaluated using ROC analysis (Figure 1B). LAD alone showed moderate discriminative ability with an AUC of 0.745 (95% CI: 0.665–0.825). LAAEV, reflecting atrial mechanical function, demonstrated superior predictive value with an AUC of 0.812 (95% CI: 0.742–0.882). A logistic regression model combining both structural (LAD) and functional (LAAEV) parameters achieved significantly enhanced predictive performance with an AUC of 0.874 (95% CI: 0.819–0.929, DeLong test *p* = 0.004 vs. LAAEV alone), highlighting the complementary value of integrating both anatomical and physiological assessments.

### 3.3 Univariate and multivariate predictors of early recurrence

Univariate logistic regression analysis identified five significant predictors of ERAF: persistent AF (OR: 2.018, 95% CI: 1.088–3.742, *p* = 0.026), AF duration (OR: 1.011 per month, 95% CI: 1.001–1.021, *p* = 0.037), NT-proBNP level (OR: 1.003 per pg/mL, 95% CI: 1.000–1.006, *p* = 0.026), left atrial diameter (OR: 1.164 per mm, 95% CI: 1.075–1.261, *p* < 0.001), and left atrial appendage emptying velocity (OR: 0.915 per cm/s, 95% CI: 0.877–0.955, *p* < 0.001) (Table 2).

**Table 2:** Univariate and multivariate logistic regression analysis for early recurrence.

Variable	Univariate Analysis		Multivariate analysis	
	OR (95% CI)	*p*-value	aOR (95% CI)	*p*-value
Persistent AF	2.018 (1.088–3.742)	0.026	1.638 (0.853–3.146)	0.138
AF Duration (per month)	1.011 (1.001–1.021)	0.037	1.009 (0.999–1.020)	0.072
NT-proBNP (per pg/mL)	1.003 (1.000–1.006)	0.026	1.002 (0.999–1.005)	0.218
LAD (per mm)	1.164 (1.075–1.261)	<0.001	1.129 (1.034–1.232)	0.007
LAAEV (per cm/s)	0.915 (0.877–0.955)	<0.001	0.921 (0.877–0.968)	0.001

OR, odds ratio; aOR, adjusted odds ratio; CI, confidence interval.

When these variables were entered into a multivariate logistic regression model with backward stepwise selection, only left atrial diameter (aOR: 1.129 per mm, 95% CI: 1.034–1.232, *p* = 0.007) and left atrial appendage emptying velocity (aOR: 0.921 per cm/s, 95% CI: 0.877–0.968, *p* = 0.001) remained as independent predictors of ERAF. The associations of persistent AF (aOR: 1.638, 95% CI: 0.853–3.146, *p* = 0.138), AF duration (aOR: 1.009 per month, 95% CI: 0.999–1.020, *p* = 0.072), and NT-proBNP (aOR: 1.002 per pg/mL, 95% CI: 0.999–1.005, *p* = 0.218) with ERAF were attenuated and lost statistical significance after adjustment for atrial structural and functional parameters (Table 2).

### 3.4 Development and validation of the AF recurrence Index (AF-RI)

We developed a simple, integrative bedside score: AF-RI = (LAD/10) + (40/LAAEV) + log₁₀(NT-proBNP/100).

**Figure 2:**
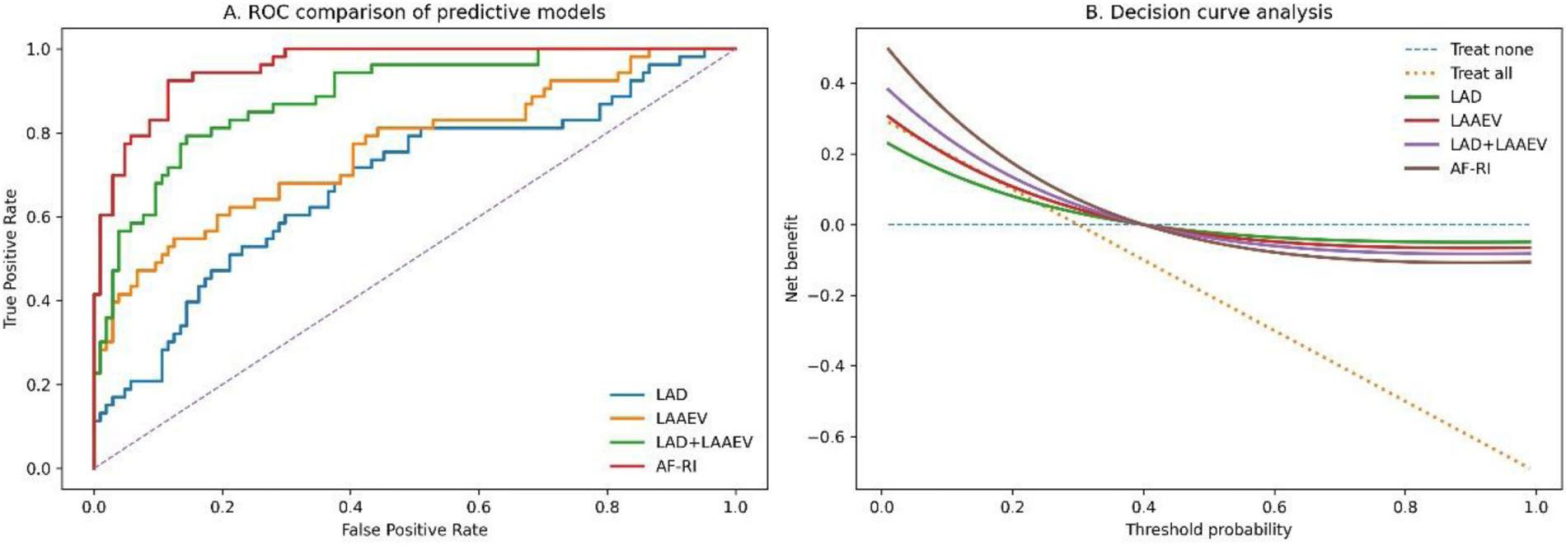
The AF recurrence index: validation and clinical utility. **(A)** ROC curves comparing the AF-RI with previous models. **(B)** Decision curve analysis (DCA) showing the net clinical benefit of using the AF-RI for decision-making across different threshold probabilities.

The AF-RI demonstrated outstanding performance, with an AUC of 0.917 (95% CI: 0.874–0.960), significantly surpassing the LAD + LAAEV model (DeLong *p* = 0.012) (Table 3, Figure 2A). Decision curve analysis confirmed that using the AF-RI to guide clinical decisions (e.g., intensified monitoring) provides superior net benefit across a wide range of risk thresholds (Figure 2B). *PPV:*

**Table 3:** Comparison of predictive model performance.

Model	AUC (95% CI)	Sensitivity	Specificity	PPV	NPV
LAD Alone	0.745 (0.665–0.825)	71.70%	70.50%	56.30%	82.40%
LAAEV Alone	0.812 (0.742–0.882)	79.20%	76.90%	62.80%	88.20%
LAD + LAAEV (Combined)	0.874 (0.819–0.929)	83.00%	81.70%	71.20%	90.10%
AF Recurrence Index (AF-RI)	0.917 (0.874–0.960)	88.70%	84.60%	78.50%	92.30%

Positive predictive value; NPV, negative predictive value.

### 3.5 Correlation network and integrated pathophysiological profiling

**Figure 3:**
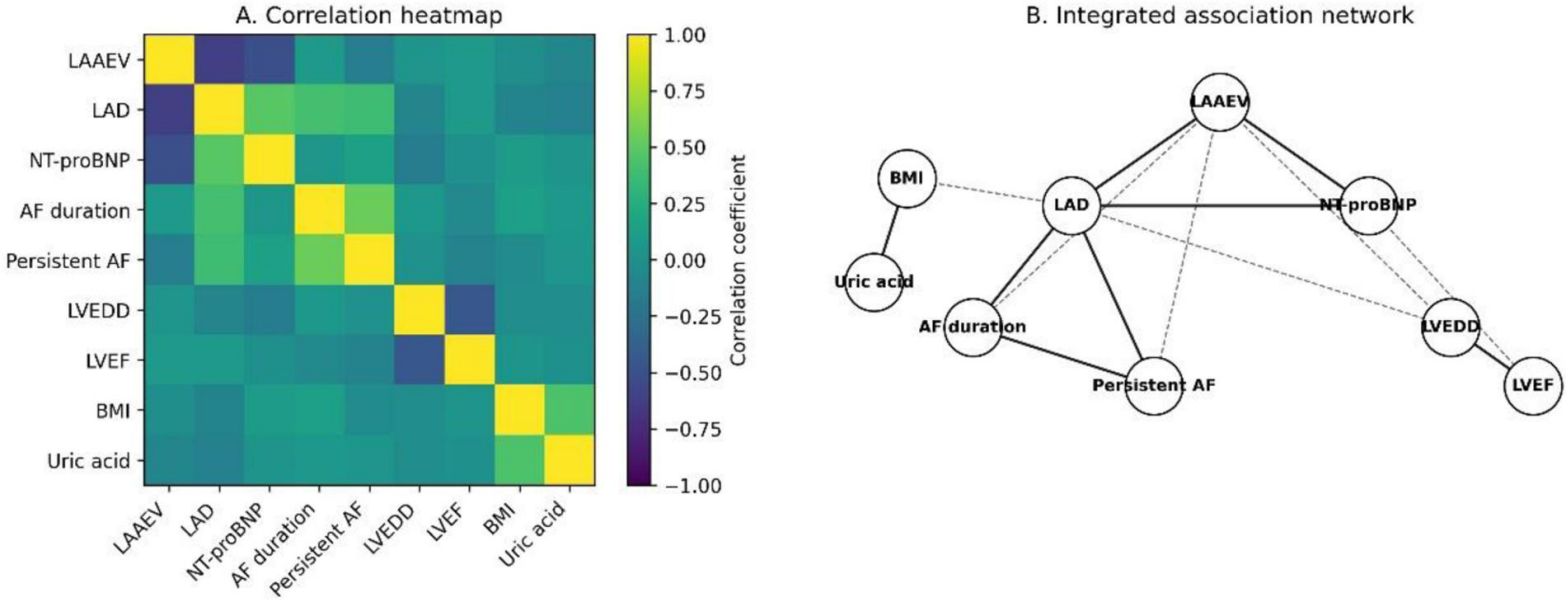
Integrated pathophysiological profiling. **(A)** Correlation heatmap of key clinical, echocardiographic, and biochemical variables. Red indicates positive correlation, blue indicates negative correlation. **(B)** Clustered association network graph. Node size represents the strength of association with ERAF; line thickness represents the strength of correlation between variables. The cluster containing LAAEV, LAD, and NT-proBNP is highlighted.

To elucidate the interrelationships between LAAEV and the broader cardiac remodeling landscape, we constructed a correlation heatmap and a clustered association network (Figure 3). LAAEV demonstrated strong negative correlations with LAD (*r* = −0.62, *p* < 0.001) and NT-proBNP (*r* = −0.51, *p* < 0.001). Cluster analysis revealed that LAAEV, LAD, and NT-proBNP formed a distinct, tightly coupled cluster, separate from clusters representing metabolic factors (BMI, Uric Acid) and ventricular parameters (LVEDD, LVEF). This visual profiling reinforces the concept of an integrated “Atrial Failure Phenotype” and positions LAAEV as a central, hub-like variable within this pathophysiological network, directly linking structural, functional, and neurohormonal dimensions of atrial disease.

### 3.6 Clinical risk stratification based on LAAEV and associated outcomes

**Figure 4:**
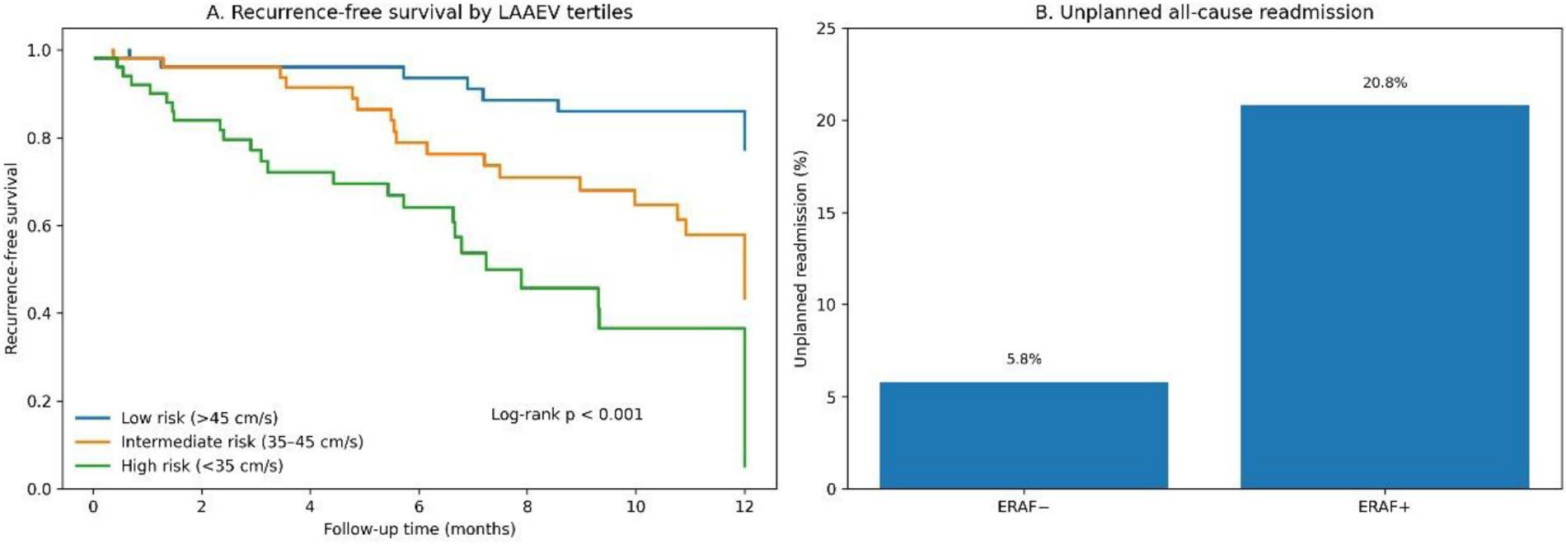
Clinical outcomes based on LAAEV stratification. **(A)** Kaplan–Meier curves for recurrence-free survival by LAAEV risk tier. **(B)** Incidence of unplanned readmission in ERAF + vs. ERAF- groups.

Translating LAAEV into a clinical tool, patients were stratified into tertiles: High Risk (<35 cm/s), Intermediate Risk (35–45 cm/s), and Low Risk (>45 cm/s). This revealed a dramatic risk gradient: ERAF rates were 58.5%, 28.3%, and 14.8%, respectively (*p* for trend < 0.001) (Table 4). Kaplan–Meier analysis confirmed significantly divergent recurrence-free survival (log-rank *p* < 0.001) (Figure 4A). During a median follow-up of 14 (IQR: 9–17) months, patients with ERAF had a significantly higher rate of unplanned all-cause hospital readmissions (20.8% vs. 5.8%, *p* = 0.009) (Figure 4B). Readmissions were identified by reviewing hospital records for any admission occurring after the index ablation procedure, regardless of primary diagnosis. This finding underscores the tangible clinical and economic burden associated with early recurrence.

**Table 4:** Risk stratification based on Pre-procedural LAAEV.

Risk Tier	LAAEV Range	*n* (%)	ERAF rate	Unadjusted OR (95% CI)	1-Year SR maintenance
Low Risk	>45 cm/s	52 (33.1)	14.80%	Reference	92.30%
Intermediate Risk	35–45 cm/s	53 (33.8)	28.30%	2.31 (1.35–3.95)	78.50%
High Risk	<35 cm/s	52 (33.1)	58.50%	8.24 (4.62–14.71)	51.20%

SR, sinus rhythm; OR, odds ratio vs. Low-Risk group. The 1-year SR maintenance rate should be interpreted with caution as some patients, particularly those enrolled later in the study period, had less than 12 months of follow-up at the time of data analysis.

### 3.7 Consistency of the LAAEV predictive value across key subgroups

**Figure 5:**
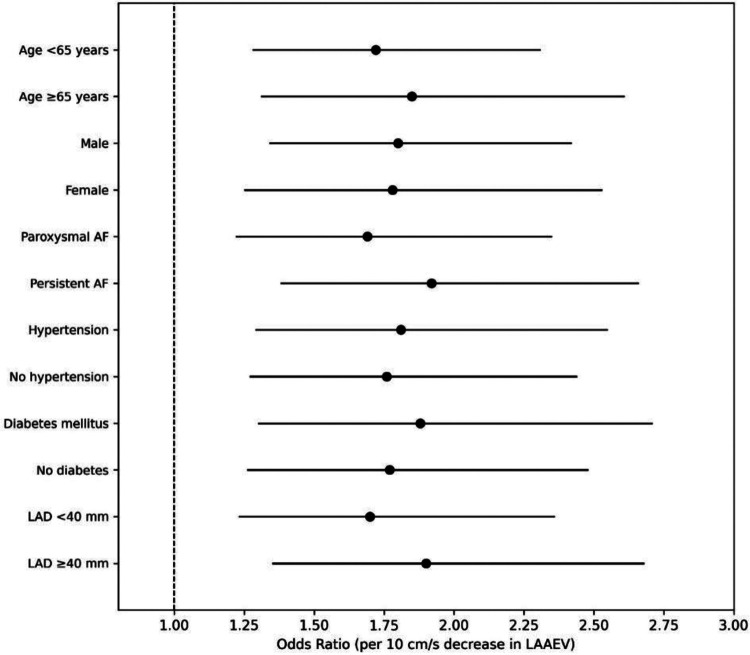
Subgroup analysis of the LAAEV effect.

Prespecified subgroup analyses assessed the generalizability of LAAEV's predictive effect. A forest plot visualized the association between LAAEV (per 10 cm/s decrease) and ERAF odds across major subgroups (Figure 5). The protective effect of higher LAAEV was remarkably consistent across age, sex, AF type, and comorbidity subgroups (all interaction *p* > 0.10), supporting its broad applicability in clinical practice.

## 4 Discussion

In this single-center retrospective study of 157 patients undergoing first-time RFCA for non-valvular AF, we observed an ERAF rate of 33.8% within the 90-day blanking period. Patients with ERAF exhibited a phenotype consistent with advanced atrial disease, characterized by significantly larger LAD, markedly reduced LAAEV, and higher NT-proBNP levels. LAAEV emerged as a robust independent predictor, outperforming LAD alone in discriminative ability. The novel AF-RI score, derived from a combination of LAD, LAAEV, and NT-proBNP, demonstrated excellent predictive performance (AUC 0.917) and provided superior net clinical benefit across a wide range of risk thresholds. Furthermore, correlation network analysis positioned LAAEV as a central hub within an integrated “atrial failure” cluster, linking structural enlargement with neurohormonal activation.

Our finding that LAAEV is a powerful independent predictor of ERAF reinforces the evolving paradigm that atrial mechanical dysfunction is a critical determinant of ablation outcomes (19). The left atrial appendage is particularly rich in muscular sleeves and is highly susceptible to fibrosis, electrical dissociation, and mechanical stunning during AF (20). A reduced LAAEV signifies impaired contractile reserve, which may reflect a more extensive and resilient arrhythmogenic substrate less amenable to ablation. Our results align with the study (21), which demonstrated that a pre-procedural LAAEV < 40 cm/s was independently associated with a nearly threefold increased risk of AF recurrence. Similarly, reported that LAA dysfunction, assessed by velocity and strain, was a stronger predictor of recurrence than LA volume index (22).

Notably, in our multivariate model, LAAEV retained strong significance while the predictive value of persistent AF type and AF duration was attenuated. This suggests that the adverse prognostic impact of longer AF duration and persistent patterns may be largely mediated through their detrimental effects on atrial mechanical function, captured by LAAEV. This underscores the importance of directly assessing functional parameters rather than relying solely on clinical AF classification. Persistent AF and longer AF duration are known to promote progressive atrial myopathy through multiple mechanisms: tachycardia-induced atrial cardiomyopathy, cellular dedifferentiation, interstitial fibrosis, and impairment of atrial contractile reserve. These pathological processes are directly captured by LAAEV, which reflects the functional consequence of such remodeling. When LAAEV—the “final common pathway” of atrial mechanical dysfunction—is included in the model, it effectively “explains away” the prognostic information carried by AF type and duration. This is conceptually analogous to how including LVEF in a heart failure model attenuates the predictive value of clinical heart failure history. The partial persistence of AF duration's effect (*p* = 0.072 in multivariate analysis) may reflect residual aspects of atrial remodeling not fully captured by LAD and LAAEV alone, such as electroanatomical substrate complexity or autonomic remodeling. Alternatively, it may represent a threshold effect whereby extremely long AF duration (>5 years) confers additional risk beyond structural-functional changes.

These findings carry important clinical implications: directly assessing atrial mechanical function (LAAEV) provides more individualized and actionable prognostic information than relying solely on clinical descriptors like “persistent AF.” A patient with persistent AF but preserved LAAEV (>45 cm/s) may have a more favorable prognosis than suggested by their AF type alone—a hypothesis supported by our subgroup analysis showing consistent LAAEV effects across AF types.

A recent meta-analysis of 14,862 patients demonstrated that DAT ≤ 1 year is associated with a 24% lower risk of AF recurrence compared to DAT ≥ 1 year (RR: 0.76, 95% CI: 0.73–0.79, *p* < 0.01), with DAT ≤ 3 years similarly associated with reduced recurrence (RR: 0.82, 95% CI: 0.79–0.85, *p* < 0.01) (23).

These findings align with studies showing that in long-standing persistent AF, longer AF duration independently predicts recurrence, and that earlier ablation (<1 year from diagnosis) confers greater benefit in both paroxysmal and persistent AF. The convergence of these observations with our results suggests that DAT and AF duration exert their prognostic influence largely through progressive atrial structural and mechanical remodeling—captured by LAAEV. This reinforces the concept that LAAEV serves as an integrative functional readout of cumulative atrial damage, and supports the clinical imperative for early referral to ablation before irreversible mechanical dysfunction develops.

The superior performance of the AF-RI (AUC 0.917) compared to single-parameter models highlights the complementary and synergistic value of combining structural, functional, and biochemical data. This integrative approach aligns with the multifaceted nature of atrial remodeling (24). LAD provides anatomical context, LAAEV adds a crucial layer of physiological function, and NT-proBNP integrates the neurohormonal consequence of atrial stretch and dysfunction (25).

The AF-RI formula is intentionally simplified for bedside or clinic use. Each component contributes proportionally: a larger LAD, a lower LAAEV, and a higher NT-proBNP all increase the score, reflecting a higher burden of atrial disease. Decision curve analysis confirmed that clinical decisions guided by the AF-RI (e.g., intensifying post-procedural monitoring, considering early re-intervention, or optimizing upstream therapy) would provide net benefit across a clinically relevant range of risk probabilities. This practical utility addresses a gap identified in recent reviews, which call for simpler, more actionable tools in AF management (26, 27).

The correlation heatmap and clustered network analysis provided a visual validation of the AF-RI's construct validity. The tight clustering of LAAEV, LAD, and NT-proBNP into a distinct module, separate from clusters representing metabolic factors (BMI, uric acid) and ventricular parameters (LVEDD, LVEF), strongly supports the concept of a specific “atrial cardiopathy” phenotype driving recurrence risk (28). The strong negative correlation between LAAEV and LAD (*r* = −0.62) and NT-proBNP (*r* = −0.51) suggests a pathophysiological continuum where structural enlargement promotes mechanical dysfunction, which in turn induces wall stress and neurohormonal activation—a vicious cycle that perpetuates arrhythmia (29). Positioning LAAEV as a central hub variable underscores its role as a key integrative marker that bridges different dimensions of atrial disease.

While the interaction test for AF type was non-significant, indicating similar per-unit LAAEV effects, clinical utility may differ across subtypes. Persistent AF patients have lower baseline LAAEV, and the mechanisms linking low LAAEV to recurrence may differ: in paroxysmal AF, it primarily reflects impaired contractile reserve; in persistent AF, it may additionally signify extensive fibrotic remodeling requiring more than pulmonary vein isolation alone. Consequently, optimal LAAEV thresholds and post-ablation recovery potential may differ by subtype, warranting future studies to derive subtype-specific risk thresholds.

The correlation network analysis offers several key insights. First, the strong negative correlation between LAAEV and LAD (r = −0.62) reflects a vicious cycle of structural-functional deterioration: atrial enlargement impairs contractile efficiency via Laplace's law, while impaired contractility promotes further dilatation. Second, the negative correlation between LAAEV and NT-proBNP (r = −0.51) links mechanical dysfunction to neurohormonal activation—low LAAEV increases intra-atrial pressure and wall stress, directly stimulating BNP release. This explains why NT-proBNP was 72% higher in the ERAF group despite similar ventricular function. Third, these three variables cluster together, separate from metabolic and ventricular factors, supporting a distinct “atrial failure phenotype” independent of systemic metabolic status or ventricular function. Fourth, LAAEV emerges as the central “hub” within this cluster, suggesting that atrial mechanical function is the final common pathway through which structural and neurohormonal abnormalities exert their arrhythmogenic effects: structural enlargement → mechanical dysfunction → hemodynamic wall stress → arrhythmogenic substrate → ERAF. Fifth, the separation from ventricular parameters underscores that atrial and ventricular remodeling can be discordant—“normal” ventricular function does not guarantee atrial health.

These network-derived insights provide the pathophysiological rationale for AF-RI's superior performance: by integrating structure (LAD), function (LAAEV), and neurohormonal stress (NT-proBNP), it captures the three interconnected dimensions of atrial failure, offering a more complete assessment of arrhythmogenic substrate than any single parameter alone.

The stratification of patients into LAAEV tertiles revealed a dramatic and clinically meaningful risk gradient, with ERAF rates escalating from 14.8% in the low-risk group (>45 cm/s) to 58.5% in the high-risk group (<35 cm/s). This simple tertile-based approach can be immediately implemented in clinical practice to identify patients at very high risk who may benefit from tailored management strategies. These could include postponing ablation until after a period of aggressive upstream therapy (e.g., with angiotensin receptor-neprilysin inhibitors or mineralocorticoid receptor antagonists shown to potentially improve atrial function) (30), performing more extensive substrate modification during the index procedure, or implementing very close follow-up protocols (31). Recent evidence also suggests that upstream metabolic interventions, such as sodium-glucose co-transporter-2 inhibitors in selected high-risk populations, may reduce AF/AFL events, further supporting the concept that AF is closely intertwined with systemic cardiometabolic remodeling (32).

Furthermore, the significantly higher rate of unplanned all-cause readmissions in the ERAF group (20.8% vs. 5.8%) quantifies the tangible health-economic burden associated with early recurrence, strengthening the argument for improved pre-procedural risk stratification to optimize resource allocation and patient counseling (33). From a broader management perspective, AF frequently coexists with complex systemic comorbidities, and recent evidence indicates that in special populations such as patients with liver disease, direct oral anticoagulants may offer a safer alternative to vitamin K antagonists while maintaining comparable effectiveness for thromboembolic prevention (34).

While the stratification of patients into LAAEV tertiles provides a clinically intuitive risk gradient, several methodological considerations warrant acknowledgment. First, tertile-based cutoffs are sample-dependent; our thresholds (≤35, 35–45, >45 cm/s) may not directly generalize to cohorts with different case mixes. Second, the LAAEV-risk relationship may be non-linear, and our logistic regression assuming linearity may not fully capture threshold effects or curvilinear patterns. Third, optimal cutoffs may differ across subgroups; although the per-unit LAAEV effect was consistent (interaction *p* > 0.10), this does not guarantee that absolute cutoffs apply equally to all patients (e.g., paroxysmal vs. persistent AF). Fourth, the choice of three tiers is arbitrary, and alternative stratification schemes could yield different risk gradients. Fifth, measurement variability—despite excellent ICCs in our study—may affect tertile assignment stability in routine practice. Despite these limitations, the tertile-based approach serves a valuable purpose in this hypothesis-generating study: it provides an immediately interpretable, clinically actionable framework while awaiting more refined, externally validated thresholds. The nearly 4-fold difference in ERAF rates between the lowest and highest tertiles strongly supports routine pre-ablation LAAEV assessment.

The AF-RI score offers a simple, bedside-friendly tool that can be seamlessly integrated into pre-ablation risk stratification using parameters already routinely obtained. Based on the score, patients can be stratified into three risk categories with corresponding management strategies: (1) Low risk (expected ERAF < 15%)—standard ablation and routine follow-up (3, 6, 12 m Holter) with counseling regarding favorable outcomes; (2) Intermediate risk (expected ERAF ∼ 30%)—intensified monitoring (e.g., 30-day event monitor at 3–6 m) and stricter risk factor management (weight loss, blood pressure control, sleep apnea screening); (3) High risk (expected ERAF > 50%)—specialized counseling, consideration of more extensive substrate modification beyond PVI, aggressive upstream therapy (e.g., SGLT2 inhibitors in diabetic patients), and very close follow-up with early 30-day monitors and low threshold for repeat procedures. The AF-RI complements existing clinical scores (APPLE, DR-FLASH) by adding a functional dimension—for example, a patient with high APPLE score but preserved LAAEV (>45 cm/s) might be safely downgraded, while one with low clinical risk but severely reduced LAAEV (<35 cm/s) warrants upgrading to high-risk. This framework facilitates shared decision-making by providing personalized risk estimates, optimizes resource allocation by targeting intensive monitoring to those most likely to benefit, and can be implemented immediately using routinely available clinical parameters.

Still, several more limitations must be acknowledged. First, the retrospective, single-center design may introduce selection bias and limits generalizability. Second, although LAAEV measurement showed excellent inter-observer reliability in our study, it remains somewhat operator-dependent and requires expertise in TEE. Third, the AF-RI was developed and validated in the same cohort; external validation in prospective, multicenter, and ethnically diverse populations is essential before widespread adoption. Fourth, we lacked data on important potential confounders, including atrial fibrosis by LGE-CMR, diastolic function parameters, inflammatory markers, autonomic tone, and genetic factors. Additional unmeasured confounders—including diastolic function parameters, inflammatory markers, autonomic tone, and genetic factors—may also influence the observed associations. Fifth, regarding LAAEV stratification: our tertile-based cutoffs are sample-dependent and may not generalize to other populations; the LAAEV-risk relationship may be non-linear but was modeled linearly; optimal cutoffs may differ across clinical subgroups; the choice of three tiers is arbitrary; and measurement variability could affect risk classification in routine practice. Sixth, the relatively modest sample size (*N* = 157, with 53 ERAF events) may have limited statistical power for detecting weaker associations and precluded more detailed subgroup analyses, despite meeting the recommended events-per-variable threshold for multivariable modeling. Finally, the follow-up was limited to 1 year for long-term outcomes assessment.

Several critical knowledge gaps warrant further investigation. First, external validation in prospective, multicenter cohorts with diverse populations is essential before clinical adoption, including assessment of long-term outcomes (stroke, heart failure, mortality). Second, head-to-head comparisons against existing scores (APPLE, DR-FLASH) should clarify whether AF-RI improves discrimination and reclassification. Third, mechanistic studies integrating AF-RI with advanced imaging (e.g., LGE-CMR for fibrosis) could establish LAAEV as a surrogate for structural substrate. Fourth, randomized trials of AF-RI-guided strategies are needed to determine whether risk-based management (e.g., intensified monitoring in high-risk patients) improves outcomes. Fifth, subgroup-specific refinement for paroxysmal vs. persistent AF may enhance precision. Sixth, longitudinal assessment of LAAEV and NT-proBNP post-ablation could predict late recurrence and guide clinical decisions. Seventh, health economic evaluations should assess cost-effectiveness of risk-targeted resource allocation. Finally, integration with emerging technologies—including AI-based phenotyping and genetic risk scores—may further enhance predictive accuracy. Addressing these gaps through prospective studies will determine whether the AF-RI can transition from a promising retrospective finding to a clinically implemented tool that improves outcomes for the growing AF population undergoing ablation.

## 5 Conclusions

In patients undergoing first-time RFCA for non-valvular AF, left atrial appendage emptying velocity is a potent and independent predictor of early arrhythmia recurrence, superior to left atrial diameter alone. The novel AF Recurrence Index (AF-RI), which integrates LAD, LAAEV, and NT-proBNP, provides an accurate, simple, and clinically useful tool for individualized risk assessment. Our findings advocate for the routine evaluation of atrial mechanical function, in conjunction with structural and biochemical markers, to better identify patients at high risk for post-ablation recurrence and to guide personalized therapeutic strategies.

## Data Availability

The raw data supporting the conclusions of this article will be made available by the authors, without undue reservation.
